# Association of Selected Genetic Variants in CYP1A1, CYP2D6, NAT1 and NAT2 with Endometrial Cancer Risk: A Preliminary Case–Control Study

**DOI:** 10.3390/ijms27135747

**Published:** 2026-06-25

**Authors:** Maciej Skrzypek, Monika Gogolewska, Andrzej Bieńkiewicz, Katarzyna Wójcik-Krowiranda, Ireneusz Majsterek, Jacek Kabziński

**Affiliations:** 1 Department of Clinical Chemistry and Biochemistry, Medical University of Lodz, 92-215 Lodz, Poland; maciej.skrzypek@umed.lodz.pl (M.S.); monikamaczynska95@gmail.com (M.G.); ireneusz.majsterek@umed.lodz.pl (I.M.); 2 Clinical Department of Gynecologic Oncology, Faculty of Health Sciences, Medical University of Lodz, 92-213 Lodz, Poland; andrzej.bienkiewicz@umed.lodz.pl (A.B.); katarzyna.wojcik-krowiranda@umed.lodz.pl (K.W.-K.)

**Keywords:** NAT1, NAT2, CYP1A1, CYP2D6, uterine cancer, SNP, xenobiotic metabolism

## Abstract

Cancer risk may be influenced by genetic variation and altered expression of xenobiotic-metabolizing enzymes, yet their role in endometrial cancer remains incompletely understood. This study evaluated the association between four polymorphisms in xenobiotic metabolism-related genes *CYP1A1* rs1799814, *CYP2D6* rs3892097, *NAT1* rs72554606, and *NAT2* rs1799930 and the risk of endometrial cancer, and assessed *CYP1A1* and *CYP2D6* expression in tumor and control tissues. Genetic association analyses, including multivariate and histology-stratified models, were performed, and gene expression levels were compared between cancer and control tissues. Variants in *NAT2*, *CYP1A1*, and *CYP2D6* were significantly associated with an increased risk of endometrial cancer, whereas *NAT1* rs72554606 showed a protective effect, particularly in the dominant model. The strongest association was observed for *NAT2* rs1799930 in additive and recessive models. Expression analysis revealed significantly higher *CYP1A1* and *CYP2D6* levels in tumor tissues than in control tissues. Stratified analyses showed generally consistent effects, especially for endometrioid carcinoma, although estimates for the serous subtype were limited by sample size. These findings suggest that polymorphisms and altered expression of xenobiotic-metabolizing genes may contribute to endometrial carcinogenesis. Further studies, including independent validation and analyses of gene–environment interactions, are needed.

## 1. Introduction

Cancer is one of the leading causes of death worldwide, and its development is the result of complex interactions between genetic and environmental factors [1]. Uterine cancer (UC), particularly endometrial cancer, is one of the most common cancers in women, and its incidence is steadily increasing, which is linked to several factors, including the aging population and the increasing prevalence of obesity [2,3]. This disease primarily affects postmenopausal women, and its etiology includes hormonal, metabolic, and genetic factors. One of the important mechanisms influencing cancer risk is the metabolism of xenobiotics, such as drugs, environmental pollutants, or dietary components [4,5]. The detoxification process of xenobiotics involves xenobiotic-metabolizing enzymes (XMEs), which are divided into phase I and phase II enzymes. Phase I enzymes, including cytochromes P450 (CYP), are responsible for the activation and modification of chemical compounds, while phase II enzymes, such as N-acetyltransferases (NATs), participate in their further detoxification and elimination [6].

Genetic variation within the genes encoding XMEs, particularly single-nucleotide polymorphisms (SNPs), can lead to changes in enzymatic activity and thus affect the body’s ability to neutralize potential carcinogens. Consequently, this may modulate individual cancer risk [7]. Polymorphisms in the *CYP1A1* and *CYP2D6* genes, which participate in the metabolism of environmental compounds, drugs, and steroid hormones, as well as in the *NAT1* and *NAT2* genes, responsible for the acetylation of aromatic amines, are particularly important [8,9].

In addition to changes at the DNA sequence level, changes in gene expression may also be a significant mechanism influencing the carcinogenesis process [10]. Abnormalities in the expression of xenobiotic-metabolizing enzymes can lead to increased activation of procarcinogens or impaired detoxification, which promotes the accumulation of DNA damage and cancer progression [11]. However, the relationship between genetic variants and the expression levels of these genes in endometrial cancer remains poorly understood.

This study analyzed four polymorphisms in genes associated with xenobiotic metabolism: *CYP1A1* rs1799814, *CYP2D6* rs3892097, *NAT1* rs72554606, and *NAT2* rs1799930. The aim of this study was to assess the association of these genetic variants with the risk of endometrial cancer, as well as to analyze the expression of the *CYP1A1* and *CYP2D6* genes in tumor and control tissues to determine their potential functional role in the pathogenesis of the disease.

## 2. Results

### 2.1. Study Population Characteristics

A total of 608 women were included in the study, comprising 308 patients with uterine cancer (UC) and 300 controls. The mean age was significantly higher in the UC group compared to controls (64.5 ± 6.4 vs. 61.1 ± 7.4 years, *p* < 0.001). The proportion of postmenopausal women was also significantly higher among UC patients (97.1%) than in the control group (91.3%, *p* = 0.004). Among patients with UC, the predominant histological subtype was endometrioid carcinoma (76.9%), followed by serous carcinoma (13.6%) and other subtypes (9.4%). Gene expression analysis was performed in 204 samples (102 cancer and 102 control). Detailed characteristics of the study population are presented in Table 1.

### 2.2. Genotype Distributions and HWE

Genotype and allele distributions for all analyzed polymorphisms are presented in Table 2. All genotype distributions in the control group were consistent with Hardy–Weinberg equilibrium (HWE), with no significant deviations observed. The observed allele frequencies were broadly comparable to those reported for European populations in publicly available databases (e.g., 1000 Genomes, gnomAD), although minor differences may reflect population-specific variation and sample size.

### 2.3. Association Between SNPs and UC Risk

**Figure 1:**
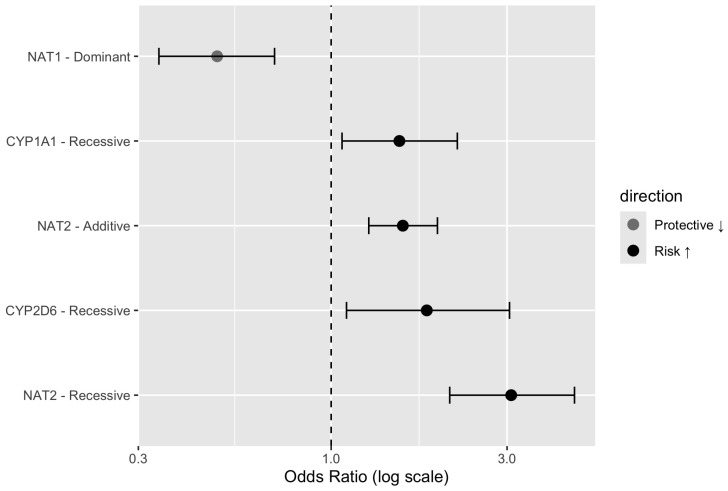
Association of polymorphisms in xenobiotic-metabolizing genes with UC risk. Whiskers represent 95% confidence intervals.

The associations between the analyzed polymorphisms and UC risk are presented in Table 3 and Figure 1. Due to significant differences in age and menopausal status between cases and controls, adjusted logistic regression models were applied. For the *NAT2* rs1799930 polymorphism, a significant association with increased UC risk was observed under the additive and recessive models. In the adjusted analysis, each additional risk allele was associated with higher odds of UC (OR = 1.56, 95% CI: 1.26–1.94, *p* < 0.001), while individuals with the homozygous variant genotype had over a threefold increased risk (OR = 3.08, 95% CI: 2.10–4.57, *p* < 0.001).

In contrast, the *NAT1* rs72554606 polymorphism was associated with a reduced risk of UC. The dominant model showed a significant protective effect (OR = 0.49, 95% CI: 0.34–0.70, *p* < 0.001), which was also observed in the additive model (OR = 0.68, 95% CI: 0.54–0.85, *p* < 0.001).

For the *CYP1A1* rs1799814 polymorphism, a significant association with increased UC risk was found in the recessive model (OR = 1.53, 95% CI: 1.07–2.20, *p* = 0.020), whereas no significant associations were observed in the additive or dominant models.

Similarly, the *CYP2D6* rs3892097 polymorphism showed a significant association with increased UC risk in the recessive model (OR = 1.82, 95% CI: 1.10–3.05, *p* = 0.021), while the additive and dominant models were not statistically significant.

### 2.4. Stratified Analysis by Histological Subtype

Stratified analyses according to histological subtype are presented in Table 4.

In patients with endometrioid carcinoma, the associations observed in the overall analysis were largely maintained. The *NAT2* rs1799930 polymorphism was significantly associated with increased risk under both additive (OR = 1.59, 95% CI: 1.27–2.01, *p* < 0.001) and recessive models (OR = 3.22, 95% CI: 2.14–4.85, *p* < 0.001). Similarly, the *NAT1* rs72554606 polymorphism showed a protective effect in the additive (OR = 0.67, 95% CI: 0.52–0.85, *p* = 0.001) and dominant models (OR = 0.49, 95% CI: 0.33–0.72, *p* < 0.001).

For *CYP1A1* rs1799814, a significant association was observed in the recessive model (OR = 1.58, 95% CI: 1.08–2.32, *p* = 0.020), while for *CYP2D6* rs3892097 a borderline association was noted in the recessive model (OR = 1.71, 95% CI: 1.00–2.95, *p* = 0.051).

In the serous subtype, similar trends were observed, although based on smaller sample sizes. The *NAT2* rs1799930 polymorphism remained significantly associated with increased risk in both additive (OR = 1.69, 95% CI: 1.08–2.66, *p* = 0.023) and recessive models (OR = 2.55, 95% CI: 1.24–5.24, *p* = 0.011). A significant association was also observed for *CYP1A1* rs1799814 in the dominant model (OR = 0.49, 95% CI: 0.25–0.96, *p* = 0.039), while no significant associations were found for *NAT1* or *CYP2D6* polymorphisms.

Overall, the direction of effects across histological subtypes was consistent with the main analysis; however, these findings should be interpreted with caution due to the limited sample size in subtype-specific analyses. To further explore the functional relevance of the analyzed polymorphisms, gene expression analysis of *CYP1A1* and *CYP2D6* was performed.

### 2.5. Gene Expression Analysis

**Figure 2:**
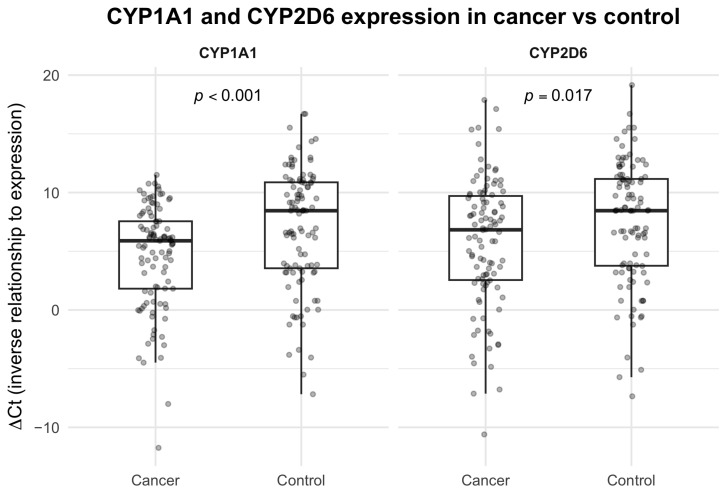
Differential expression of *CYP1A1* and *CYP2D6* between cancer and control samples.

Gene expression analysis was performed using ΔCt values (Ct_target − Ct_GAPDH), where lower ΔCt values indicate higher gene expression. *CYP1A1* expression was significantly higher in cancer samples compared to controls. The median ΔCt was 5.89 (IQR: 5.74) in cancer samples and 8.45 (IQR: 7.32) in controls (*p* < 0.001). Similarly, *CYP2D6* expression was significantly increased in cancer samples. The median ΔCt was 6.83 (IQR: 7.16) in cancer samples and 8.46 (IQR: 7.39) in controls (*p* = 0.017). Gene expression results are presented in Table 5 and Figure 2.

## 3. Discussion

This study assessed the association of four polymorphisms in xenobiotic-metabolizing genes (*NAT2* rs1799930, *NAT1* rs72554606, *CYP1A1* rs1799814, and *CYP2D6* rs3892097) with the risk of endometrial cancer. We demonstrated a significant increase in disease risk for variants in the *NAT2*, *CYP1A1*, and *CYP2D6* genes, while the *NAT1* polymorphism demonstrated a protective effect. Additionally, significantly higher expression of the *CYP1A1* and *CYP2D6* genes was observed in cancer tissues compared to control tissues. Together, these results suggest a potential role for both genetic variation and perturbations in the expression of xenobiotic-metabolizing genes in the pathogenesis of endometrial cancer. To account for potential confounding, all analyses were adjusted for age and menopausal status. However, residual confounding cannot be excluded. Age-related and hormonal influences may contribute to the observed differences; however, adjustment for these variables reduces the likelihood that the results are solely driven by these factors.

The strongest effect was observed for the *NAT2* rs1799930 polymorphism, which was significantly associated with an increased risk of endometrial cancer in both additive and recessive models. This result is consistent with the hypothesis that reduced activity of N-acetyltransferase enzymes, characteristic of so-called slow acetylators, leads to impaired detoxification of aromatic amines and other potential carcinogens [12,13]. Consequently, increased exposure of cells to genotoxic compounds may occur, promoting the accumulation of DNA damage and the initiation of the neoplastic process. These findings are consistent with previous studies indicating an association of *NAT2* variants with the risk of various cancers [14]. In contrast to *NAT2*, the *NAT1* rs72554606 polymorphism demonstrated a significant protective effect, particularly in the dominant model. The biological mechanism underlying the observed protective association of *NAT1* rs72554606 remains unclear. In the absence of functional data, any interpretation regarding its impact on enzyme activity or substrate specificity remains speculative. It should be emphasized that the results regarding *NAT1* in the literature are equivocal, suggesting that the effect of this gene on cancer risk may depend on the environmental context and the type of cancer [15].

Particularly interesting results were obtained for the *CYP1A1* and *CYP2D6* genes. Gene expression analysis was limited to *CYP1A1* and *CYP2D6* due to their key role in phase I metabolism and their previously reported involvement in carcinogen activation. The results demonstrated a significant association of their variants with endometrial cancer risk, while expression analysis revealed higher expression levels in neoplastic tissues. Cytochromes P450 play a key role in xenobiotic metabolism, including the activation of procarcinogens into reactive forms capable of DNA damage [16]. Increased expression of *CYP1A1* and *CYP2D6* may therefore lead to increased carcinogen bioactivation and disruption of steroid hormone metabolism, which may promote cancer development [17,18]. Although both genetic associations and expression differences were observed for *CYP1A1* and *CYP2D6*, the present study did not assess the relationship between genotype and gene expression. Therefore, these findings should be interpreted as complementary but independent observations rather than evidence of a direct functional link. It should be noted, however, that this study did not examine the direct relationship between individual genetic variants and gene expression levels. Therefore, it is impossible to definitively determine whether the observed expression changes are a direct consequence of the analyzed polymorphisms. However, the consistent direction of effects (increased risk and increased expression) may indicate biological coherence of the findings, although no conclusions regarding functional relationships can be drawn.

In the analysis stratified by histological type, the direction of effects was generally consistent with the results of the overall analysis, particularly for endometrioid carcinoma. Similar trends were also observed in the serous subtype, but the sample size was limited, which affects the precision of the estimates and requires cautious interpretation of the results.

Overall, the obtained results are partially consistent with previous reports on the role of xenobiotic-metabolizing genes in carcinogenesis. In particular, the association of *NAT2* variants with an increased risk of cancer is well documented [17,19,20]. Data regarding *NAT1* remain more equivocal, which may be due to population differences and gene–environment interactions. In the case of CYP genes, the results of previous studies indicate their potential involvement in neoplastic processes, but simultaneous analysis of polymorphisms and expression, as in the present study, is less frequently reported [21,22].

The obtained results suggest that polymorphisms in xenobiotic-metabolizing genes, particularly *NAT2*, *CYP1A1*, and *CYP2D6*, may be potential markers of endometrial cancer risk. Furthermore, the observed changes in CYP gene expression may have biological relevance and could potentially influence the course of the disease. In the future, combining genetic and expression data may contribute to better identification of individuals at increased risk and the development of personalized medicine strategies.

The main advantages of the study include the relatively large cohort size and a comprehensive approach encompassing both the analysis of genetic polymorphisms and gene expression. Additional advantages include the inclusion of multivariate models adjusted for relevant clinical variables, such as age and menopausal status, as well as the conduct of analyses stratified by histological type. However, the study also has certain limitations. First, the lack of an independent validation cohort limits the generalizability of the results. Second, the direct effect of polymorphisms on gene expression levels was not analyzed. Furthermore, the expression analysis was limited to two genes, which does not allow for a comprehensive assessment of metabolic pathways. Finally, the lack of data on environmental exposures, including potential contact with carcinogens or lifestyle factors such as smoking, limits the ability to assess gene–environment interactions and the combined effect of genetic and environmental risk factors. Moreover, important clinical variables such as BMI, parity, hormone replacement therapy, and tumor stage were not available for all patients, which represents a limitation and may introduce residual confounding.

In conclusion, the results of this study indicate that polymorphisms in xenobiotic-metabolizing genes, particularly *NAT2*, *CYP1A1*, and *CYP2D6*, are associated with the risk of endometrial cancer. Concurrently, increased expression of *CYP1A1* and *CYP2D6* genes in tumor tissues is consistent with their potential involvement in carcinogenesis. These data highlight the importance of xenobiotic metabolism disorders in the pathogenesis of endometrial cancer and indicate the need for further research in this area. It should be emphasized that the present analyses are exploratory in nature and do not provide direct evidence of causal or functional relationships between the analyzed polymorphisms and gene expression or enzyme activity. Furthermore, the present study has a relatively modest sample size compared to large-scale genome-wide association studies. Therefore, the findings should be considered preliminary and hypothesis-generating rather than definitive evidence of genetic associations. Further studies in larger, independent cohorts are required to validate these observations.

## 4. Materials and Methods

### 4.1. Study Population

A total of 608 women were included in the study, comprising 308 patients with histopathologically confirmed uterine cancer and 300 controls. Tumor samples were collected from the Department of Gynecological Oncology, Copernicus Memorial Hospital in Lodz, Poland. The inclusion criterion for the study group was histopathologically confirmed uterine cancer. Patients with a coexisting or previous history of other invasive malignancies were excluded.

The control group consisted of women who underwent hysterectomy for non-oncological reasons. The inclusion criterion for controls was the absence of any neoplastic disease.

All participants provided informed consent. The study was approved by the Bioethics Committee (no. RNN/273/16KE).

### 4.2. DNA Isolation and Genotyping

Genomic DNA was isolated using the Genomic Mini DNA Kit (A&A Biotechnology, Gdańsk, Poland) according to the manufacturer’s instructions. DNA concentration and purity were assessed using a Synergy HT Multi-Mode Microplate Reader (BioTek Instruments, Inc., Winooski, VT, USA).

Genotyping of four single-nucleotide polymorphisms (SNPs) in xenobiotic metabolism genes was performed using TaqMan SNP Genotyping Assays (Thermo Fisher Scientific, Waltham, MA, USA). The analyzed polymorphisms included *NAT2* rs1799930, *NAT1* rs72554606, *CYP1A1* rs1799814, and *CYP2D6* rs3892097. The selected polymorphisms were chosen based on their involvement in xenobiotic metabolism pathways, potential functional relevance, and prior evidence suggesting their association with cancer susceptibility. Additionally, the availability of validated genotyping assays and their frequency in European populations were taken into account. Notably, *CYP1A1* rs1799814 has been previously investigated in endometrial cancer; however, its combined analysis with *CYP2D6*, *NAT1*, and *NAT2* polymorphisms, together with gene expression data, has not been comprehensively assessed.

Polymerase chain reaction (PCR) was carried out according to the manufacturer’s protocol.

### 4.3. RNA Isolation and Gene Expression Analysis

Total RNA was isolated from approximately 40 mg of frozen tissue using the GeneElute Mammalian Total RNA Miniprep Kit (Sigma-Aldrich, St. Louis, MO, USA) according to the manufacturer’s instructions. RNA samples were stored at −70 °C until further analysis. RNA integrity and concentration were assessed prior to cDNA synthesis.

Complementary DNA (cDNA) was synthesized using the High-Capacity cDNA Reverse Transcription Kit (Applied Biosystems, Thermo Fisher Scientific, Waltham, MA, USA).

Gene expression analysis was performed using real-time PCR with TaqMan Universal Master Mix and TaqMan assays (*CYP1A1*: Hs01054796_g1; *CYP2D6*: Hs04931916_gH) (Applied Biosystems, Thermo Fisher Scientific, Waltham, MA, USA). GAPDH was used as an endogenous control. Reactions were performed on a CFX Connect Real-Time PCR Detection System (Bio-Rad Laboratories, Hercules, CA, USA).

Control samples for gene expression analysis consisted of non-neoplastic endometrial tissue obtained from women undergoing hysterectomy for benign conditions. Relative gene expression was calculated using the ΔCt method (Ct_target − Ct_GAPDH). All reactions were performed in duplicate.

### 4.4. Statistical Analysis

Statistical analyses were performed using R software (version 4.5.1 [23]). Continuous variables are presented as mean ± standard deviation (SD) or median (interquartile range, IQR), as appropriate, while categorical variables are presented as counts and percentages.

Normality of continuous variables was assessed using the Shapiro–Wilk test. Differences between groups were evaluated using Student’s *t*-test or the Mann–Whitney U test for continuous variables and the chi-square test for categorical variables.

Hardy–Weinberg equilibrium (HWE) was evaluated in the control group using the chi-square test.

Associations between SNPs and uterine cancer risk were assessed using logistic regression models under additive, dominant, and recessive genetic models. Both crude and multivariable-adjusted models were calculated. Adjusted models included age and menopausal status as covariates. Odds ratios (ORs) with 95% confidence intervals (CIs) were reported.

Differences in gene expression (ΔCt values) between cancer and control groups were assessed using the Mann–Whitney U test.

All statistical tests were two-sided, and a *p*-value < 0.05 was considered statistically significant. No correction for multiple testing was applied, as analyses were hypothesis-driven.

## Figures and Tables

**Table 1:** Clinical characteristics of the study population.

Variable	Cases (*n* = 308)	Controls (*n* = 300)	*p*-Value
Age, years (mean ± SD)	64.5 ± 6.4	61.1 ± 7.4	<0.001
Menopause, POST	299 (97.1%)	274 (91.3%)	0.004
Histology: Endometrioid	237 (76.9%)	-	
Histology: Serous	42 (13.6%)	-	
Histology: Other	29 (9.4%)	-	

**Table 2:** Genotype and allele frequencies of analyzed polymorphisms.

SNP	Genotype	Cases (*n* = 308); *n* (%)	Controls (*n* = 300); *n* (%)	Allele Frequency (Controls)	HWE *p*-Value
*NAT2* rs1799930	AA	100 (32.5%)	109 (36.3%)	A: 0.532/G: 0.468	0.5035
	AG	90 (29.2%)	139 (46.3%)		
	GG	118 (38.3%)	52 (17.3%)		
*NAT1* rs72554606	CC	120 (39.0%)	74 (24.7%)	C: 0.544/T: 0.456	0.8197
	CT	125 (40.6%)	148 (49.3%)		
	TT	63 (20.5%)	78 (26.0%)		
*CYP1A1* rs1799814	AA	90 (29.2%)	80 (26.7%)	A: 0.488/C: 0.512	0.2041
	AC	114 (37.0%)	139 (46.3%)		
	CC	104 (33.8%)	81 (27.0%)		
*CYP2D6* rs3892097	AA	141 (45.8%)	151 (50.3%)	A: 0.678/G: 0.322	0.4725
	AG	120 (39.0%)	120 (40.0%)		
	GG	47 (15.3%)	29 (9.7%)		

**Table 3:** Association between gene polymorphisms and uterine cancer risk (adjusted for age and menopausal status).

SNP	Model	OR (95% CI)	*p*-Value
*NAT2* rs1799930	Additive	**1.56 (1.26–1.94)**	**<0.001**
	Dominant	1.27 (0.90–1.81)	0.171
	Recessive	**3.08 (2.10–4.57)**	**<0.001**
*NAT1* rs72554606	Additive	**0.68 (0.54–0.85)**	**<0.001**
	Dominant	**0.49 (0.34–0.70)**	**<0.001**
	Recessive	0.72 (0.49–1.07)	0.106
*CYP1A1* rs1799814	Additive	1.13 (0.91–1.41)	0.258
	Dominant	0.92 (0.63–1.32)	0.639
	Recessive	**1.53 (1.07–2.20)**	**0.020**
*CYP2D6* rs3892097	Additive	1.23 (0.97–1.56)	0.084
	Dominant	1.15 (0.83–1.60)	0.393
	Recessive	**1.82 (1.10–3.05)**	**0.021**

All models were adjusted for age and menopausal status.

**Table 4:** Stratified analysis by histological subtype.

Subtype	SNP/Model	OR (95% CI)	*p*-Value
Endometrioid	*NAT2* Additive	**1.59 (1.27–2.01)**	**<0.001**
Endometrioid	*NAT2* Recessive	**3.22 (2.14–4.85)**	**<0.001**
Endometrioid	*NAT1* Dominant	**0.49 (0.33–0.72)**	**<0.001**
Endometrioid	*CYP1A1* Recessive	**1.58 (1.08–2.32)**	**0.020**
Endometrioid	*CYP2D6* Recessive	1.71 (1.00–2.95)	0.051
Serous	*NAT2* Additive	**1.69 (1.08–2.66)**	**0.023**
Serous	*NAT2* Recessive	**2.55 (1.24–5.24)**	**0.011**
Serous	*CYP1A1* Dominant	**0.49 (0.25–0.96)**	**0.039**

All models were adjusted for age and menopausal status.

**Table 5:** Summary of *CYP1A1* and *CYP2D6* expression levels in cancer and control groups based on ΔCt values.

Gene	Group	*n*	Median ΔCt	IQR ΔCt	*p*-Value
*CYP1A1*	Cancer	102	5.89	5.74	<0.001
*CYP1A1*	Control	102	8.45	7.32	
*CYP2D6*	Cancer	102	6.83	7.16	0.017
*CYP2D6*	Control	102	8.46	7.39	

## Data Availability

The original contributions presented in this study are included in the article. Further in-quiries can be directed to the corresponding author(s).

## References

[1] Bray F., Laversanne M., Sung H., Ferlay J., Siegel R.L., Soerjomataram I., Jemal A. (2024). Global Cancer Statistics 2022: GLOBOCAN Estimates of Incidence and Mortality Worldwide for 36 Cancers in 185 Countries. CA Cancer J. Clin..

[2] Felix A.S., Brinton L.A. (2018). Cancer Progress and Priorities: Uterine Cancer. Cancer Epidemiol. Biomark. Prev..

[3] Henley S.J., Miller J.W., Dowling N.F., Benard V.B., Richardson L.C. (2018). Uterine Cancer Incidence and Mortality—United States, 1999–2016. MMWR Morb. Mortal. Wkly. Rep..

[4] Joseph T., Chacko P., Wesley R., Jayaprakash P.G., James F.V., Pillai M.R. (2006). Germline Genetic Polymorphisms of CYP1A1, GSTM1 and GSTT1 Genes in Indian Cervical Cancer: Associations with Tumor Progression, Age and Human Papillomavirus Infection. Gynecol. Oncol..

[5] Williams J.A., Phillips D.H. (2000). Mammary Expression of Xenobiotic Metabolizing Enzymes and Their Potential Role in Breast Cancer. Cancer Res..

[6] Stipp M.C., Acco A. (2021). Involvement of Cytochrome P450 Enzymes in Inflammation and Cancer: A Review. Cancer Chemother. Pharmacol..

[7] Dhakshinamoorthy S., Long D.J., Jaiswal A.K. (2000). Antioxidant Regulation of Genes Encoding Enzymes That Detoxify Xenobiotics and Carcinogens. Curr. Top. Cell. Regul..

[8] Hein D.W., Doll M.A., Fretland A.J., Leff M.A., Webb S.J., Xiao G.H., Devanaboyina U.S., Nangju N.A., Feng Y., Ferguson R.J. (2000). Molecular Genetics and Epidemiology of the NAT1 and NAT2 Acetylation Polymorphisms. Cancer Epidemiol. Biomark. Prev..

[9] Androutsopoulos V.P., Tsatsakis A.M., Spandidos D.A. (2009). Cytochrome P450 CYP1A1: Wider Roles in Cancer Progression and Prevention. BMC Cancer.

[10] Beyerle J., Holowatyj A.N., Haffa M., Frei E., Gigic B., Schrotz-King P., Brenner H., Habermann N., Scherer D., Ulrich C.M. (2020). Expression Patterns of Xenobiotic-Metabolizing Enzymes in Tumor and Adjacent Normal Mucosa Tissues among Patients with Colorectal Cancer: The ColoCare Study. Cancer Epidemiol. Biomark. Prev..

[11] Rodriguez M., Potter D.A. (2013). CYP1A1 Regulates Breast Cancer Proliferation and Survival. Mol. Cancer Res..

[12] Sillanpää P., Hirvonen A., Kataja V., Eskelinen M., Kosma V.M., Uusitupa M., Vainio H., Mitrunen K. (2005). NAT2 Slow Acetylator Genotype as an Important Modifier of Breast Cancer Risk. Int. J. Cancer.

[13] Golka K., Prior V., Blaszkewicz M., Bolt H.M. (2002). The Enhanced Bladder Cancer Susceptibility of NAT2 Slow Acetylators towards Aromatic Amines: A Review Considering Ethnic Differences. Toxicol. Lett..

[14] Tian F.S., Shen L., Ren Y.W., Zhang Y., Yin Z.H., Zhou B.S. (2014). N-Acetyltransferase 2 Gene Polymorphisms Are Associated with Susceptibility to Cancer: A Meta-Analysis. Asian Pac. J. Cancer Prev..

[15] Agúndez J.A. (2008). Polymorphisms of Human N-Acetyltransferases and Cancer Risk. Curr. Drug Metab..

[16] Agúndez J.A.G. (2004). Cytochrome P450 Gene Polymorphism and Cancer. Curr. Drug Metab..

[17] Zhang Y.Y., Yang L. (2009). Interactions between Human Cytochrome P450 Enzymes and Steroids: Physiological and Pharmacological Implications. Expert Opin. Drug Metab. Toxicol..

[18] Rebbeck T.R., Su H.I., Sammel M.D., Lin H., Tran T.V., Gracia C.R., Bunin G.R., DeMichele A., Strom B.L. (2010). Effect of Hormone Metabolism Genotypes on Steroid Hormone Levels and Menopausal Symptoms in a Prospective Population-Based Cohort of Women Experiencing the Menopausal Transition. Menopause.

[19] Çayan F., Ayaz L., Aras-Ateş N., Dilekçi E., Dilek S., Tamer-Gümüş L. (2009). N-Acetyltransferase 2 Gene Polymorphism in Patients with Cervical Cancer. Int. J. Gynecol. Cancer.

[20] Bafligil C., Thompson D.J., Lophatananon A., Smith M.J., Ryan N.A., Naqvi A., Evans D.G., Crosbie E.J. (2020). Association between Genetic Polymorphisms and Endometrial Cancer Risk: A Systematic Review. J. Med. Genet..

[21] Xu J., Tan C. (2024). Interaction between CYP1A1 Gene Polymorphism and Environment Factors on Risk of Endometrial Cancer. Environ. Health Prev. Med..

[22] Spyrou I., Sifakis S., Ploumidis A., Papalampros A.E., Felekouras E., Tsatsakis A.M., Spandidos D.A. (2014). Expression Profile of CYP1A1 and CYP1B1 Enzymes in Endometrial Tumors. Tumor Biol..

[23] R Core Team (2019). R: A Language and Environment for Statistical Computing.

